# Comparative chloroplast genomes provided insights into the evolution and species identification on the Datureae plants

**DOI:** 10.3389/fpls.2023.1270052

**Published:** 2023-10-24

**Authors:** He Su, Xiaoxia Ding, Baosheng Liao, Danchun Zhang, Juan Huang, Junqi Bai, Subing Xu, Jing Zhang, Wen Xu, Xiaohui Qiu, Lu Gong, Zhihai Huang

**Affiliations:** ^1^ The Second Clinical College, Guangzhou University of Chinese Medicine, Guangzhou, China; ^2^ Key Laboratory of Quality Evaluation of Chinese Medicine of the Guangdong Provincial Medical Products Administration, Guangdong Provincial Hospital of Chinese Medicine, Guangzhou, China

**Keywords:** Datureae, chloroplast genome, comparative analysis, species identification markers, evolutionary relationship

## Abstract

Generally, chloroplast genomes of angiosperms are always highly conserved but carry a certain number of variation among species. In this study, chloroplast genomes of 13 species from Datureae tribe that are of importance both in ornamental gardening and medicinal usage were studied. In addition, seven chloroplast genomes from Datureae together with two from Solanaceae species retrieved from the National Center for Biotechnology Information (NCBI) were integrated into this study. The chloroplast genomes ranged in size from 154,686 to 155,979 and from 155,497 to 155,919 bp for species of *Datura* and *Brugmansia*, respectively. As to *Datura* and *Brugmansia*, a total of 128 and 132 genes were identified, in which 83 and 87 protein coding genes were identified, respectively; Furthermore, 37 tRNA genes and 8 rRNA genes were both identified in *Datura* and *Brugmansia.* Repeats analysis indicated that the number and type varied among species for Simple sequence repeat (SSR), long repeats, and tandem repeats ranged in number from 53 to 59, 98 to 99, and 22 to 30, respectively. Phylogenetic analysis based on the plastid genomes supported the monophyletic relationship among *Datura* and *Brugmansia* and *Trompettia*, and a refined phylogenic relationships among each individual was resolved. In addition, a species-specific marker was designed based on variation spot that resulted from a comparative analysis of chloroplast genomes and verified as effective maker for identification of *D. stramonium* and *D. stramonium* var. *inermis*. Interestingly, we found that 31 genes were likely to be under positive selection, including genes encoding ATP protein subunits, photosystem protein subunit, ribosome protein subunits, NAD(P)H dehydrogenase complex subunits, and *clp*P, *pet*B, *rbc*L, *rpo*Cl, *ycf*4, and *cem*A genes. These genes may function as key roles in the adaption to diverse environment during evolution. The diversification of Datureae members was dated back to the late Oligocene periods. These chloroplast genomes are useful genetic resources for taxonomy, phylogeny, and evolution for Datureae.

## 1 Introduction

**Figure 1:**
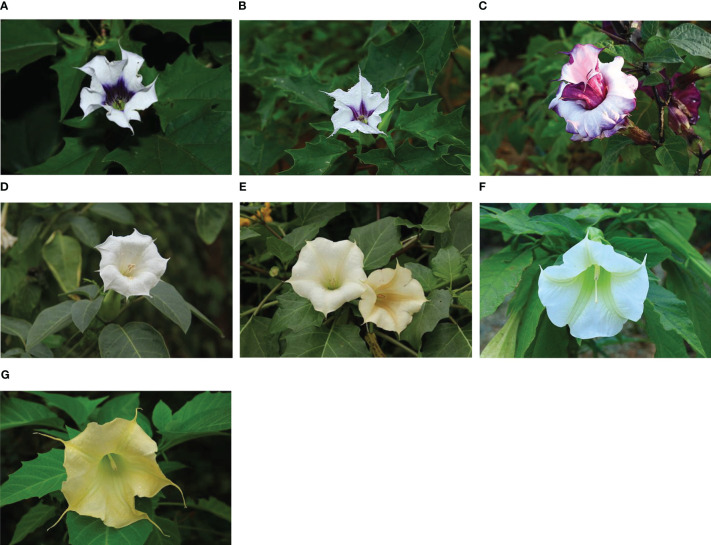
Collected species of Datureae in this study. **(A)** *D. stramonium*; **(B)** *D*. *stramonium* var. *inermis*; **(C)** *D. stramonium* var. *tatula*; **(D)** *D. inoxia*; **(E)** *D. metel*; **(F)** *B. arborea*; **(G)** *B. aurea*. Photo taken by Chongjian Zhou.

Datureae, a tribe belonging to Solanaceae, widely distributed around the world, consists of three clades: *Datura* L., *Brugmansia* Pers., and *Trompettia* gen. nov. It is of importance both in ornamental gardening for its charismatic large flowers (**Figure 1**) and in medicinal usage for its therapeutic effects in inflammations, skin infections, and rheumatic arthritis (Zhengping et al., 2003; Pigatto et al., 2015; Algradi et al., 2021). Datureae species are widely distributed around the world, while the geographical distribution varied among its members. For example, *Datura* are found in the southwest U.S.A. and Mexico and parts of central America (Dupin and Smith, 2018), while species of *Brugmansia* distribute in the Andes and southern portions of the Atlantic forest in Brazil (Chellemi et al., 2011; Kim et al., 2020). In addition, expanding the distribution of several species is also attributed to human activities. The phylogenic analysis for Datureae clade have been addressed and made it easily distinguished through a suite of morphological features. For example, *Datura* and *Brungmansia* are distinguished by phenotypes such as fruit type, fruit shape, seed shape, and seed margin (Bye and Sosa, 2013). However, revision of taxonomy and phylogeny relationships continues to be updated; a new monotypic genus, *Trompettia*, a species previously described in *Iochrom* Benth, was incorporated in Datureae that owns contorted-conduplicate corolla aestivation characteristics, leading *Trompettia* to be a new member of Datureae (Smith and Baum, 2006; Olmstead et al., 2008). In addition, the phylogeny relationships among those genus have been verified by a study combining information of morphological and three nuclear markers (Dupin and Smith, 2018). However, the reality that the majority of classification characteristics for Datureae depends on characteristics of flowers and seeds have made it hard to distinguish or identify before flowering period, leading to unpredictable disasters. For instance, some species of *Datura* and *Brugmansia*, abundant in tropane alkaloids varying in species and in dose, have been used as phytomedicines in the treatment of inflammations, skin infections, rheumatic arthritis, etc. (Berkov et al., 2006; Benítez et al., 2018; Petricevich et al., 2020; Cinelli and Jones, 2021). However, tropane alkaloids also result in insidious toxicity and may cause hallucinations and poisoning outbreaks and even death in high dose (Doan et al., 2019; Mutebi et al., 2022). Due to similar appearance, the Chinese crude drugs of these species are frequently misidentified; for example, the dried flowers of *D. metel* listed in the Chinese Pharmacopeia are always mixed with those of *D. stramonium*, *D. inoxia*, and even *B. arborea* during sale process (Han et al., 2011; Wu et al., 2015).

Although the monophyly of Datureae is not contested, relationships within and among genera are still under-addressed. For example, the phylogenetic relationships between *Datura* and other two genera are still unclear, although the phylogeny of *Datura* have been extensively studied (Luna-Cavazos et al., 2009). Some researchers regarded *T. cardenasiana* as sister to *Brugmansia* (Särkinen et al., 2013) (Särkinen et al., 2013), while others thought it as sister to *Datura* and *Brugmansia* (Ng and Smith, 2016; Dupin and Smith, 2018). High similarity in morphological characteristics among species for the sub-genus of Datureae have made it a challenge for its classification and led to confusion in commercial herbal market. Previous studies have conducted the molecular classification and identification analysis on those genus. Han et al. employed four universal DNA barcodes (ITS2, *psbA-trn*H, *mat*K, and *rbc*L) to identify *D. metel* and its adulterants (Han et al., 2011). Wu conducted the identification study of *Datura* by using the ITS2 barcode (Wu et al., 2015). However, limited resolution of Sanger sequencing has reduced the capacity of variation identification in Datureae, which limited its application in identification and phylogenetic studies of Datureae. In addition, it was found that the origin of Datureae tribe was approximately 35 million years ago, when the beginning of the Andean uplift based on three nuclear markers (ITS+5.8S, *waxy* and *lfy*) (Dupin and Smith, 2018). The uniparental inherited chloroplast (cp) genome, a functional important organelle conducting photosynthesis that converts solar energy into chemical energy and release oxygen, owing to >100 kb genome in size and is highly conserved both in genome structure and nucleotide substitution rates during evolution, has been proven to be a potential tool in species identification and evolution trajectory estimation (Brunkard et al., 2015; Li et al., 2015). For instance, Cui reported the cp genome of *Zingiber officinale* and constructed a robust phylogenetic relationship among species in the family Zingiberaceae (Cui et al., 2019). Fan identified the sequence differentiation and adaptive variation in cp genomes among the order Dipsacales and provided much useful genetic information not only for species identification but also for the understanding of evolution trajectory for Dipsacales (Fan et al., 2018). Chen showed inconsistencies between the molecular phylogeny and traditional taxonomy for subg. *Seriphidium* and found out cp genome could be used as super barcode in resolving interspecific relationships as in authentic for subg. *Seriphidium* (Chen et al., 2023). Gong et al. reported that the cp genome of *Amomum villosum*, which provided significantly higher resolution in species identification than that of ITS2 and the union of those information, gave power for the detection of hybridization event between *A. villosum* and *A. longiligulare* (Gong et al., 2022).

However, to the best of our understanding, only two species of cp genomes belonging to Datureae have been reported; more detailed studies for this tribe are still needed to be carried out (Yang et al., 2014; De-la-Cruz and Núñez-Farfán, 2020). In this study, a total of 13 complete cp genomes of Datureae were sequenced and annotated. Together with seven online available cp genomes in Datureae, we aimed to (1) study the comprehensive characteristics of Datureae cp genomes, (2) reveal the phylogenic relationships and evolutionary history among Datureae species based on cp genomes, and (3) develop the molecular markers on cp genomes for Datureae species identification. This study will facilitate the studies of genetics and evolution of Datureae species.

## 2 Materials and methods

### 2.1 Plant materials and DNA extraction

A total of 13 individuals from seven Datureae taxa were identified by Chongjian Zhou, an engineer in HuBei Guizhenyuan Chinese Herbal Medicine Co., Ltd; Huagu Ye, a professor in South China Botanical Garden, Chinese Academy of Sciences; and Jizhu Liu, a professor in Guangdong Pharmaceutical University (**Figure 1**; **Supplementary Table S1**). Fresh leaves were packaged in thin foil, then frozen by liquid nitrogen, and stored in a −80°C fridge for high throughput sequencing. Furthermore, 44 fresh samples of 13 Datureae species were used to test and verify the molecular markers (five universal DNA barcodes) (**Supplementary Tables S1**, **S2**). Genomic DNA was extracted using a DNA easy Plant Mini Kit (Qiagen Co., Hilden, Gemany) following the manufactures’ instructions. NanoDrop2000C spectrophotometry and electrophoresis in 1% (w/v) agarose gel were used to detect the concentration and integration of the total DNA, respectively.

### 2.2 Sequencing, assembly, and annotation

Extracted DNA was fragmented to an average size of approximately 400 bp using CovarisM220 (Gene Company Limited, China) for paired-end library construction. Paired-end library was constructed using NEXTFLEX® Rapid DNA-Seq (Bioo Scientific, Austin, TX, USA). Adapters containing the full complement of sequencing primer hybridization sites were ligated to the blunt end of fragments. Paired-end sequencing was performed on Illumina NovaSeq platform (Illumina Inc., San Diego, CA, USA) at Majorbio Bio-Pharm Technology Co., Ltd. (Shanghai, China). More than 4-Gb raw reads per sample were generated (**Supplementary Table S2**). Raw reads were quality controlled with Trimmomatic and Fast QC software (https://www.bioinformatics.babraham.ac.uk/projects/fastqc). The assembly strategy of cp genome referred to Zhou’s (Zhou et al., 2017). Cp-like reads were extracted by mapping clean reads against the collection of cp genomes retrieved from the NCBI nucleotide database on the basis of their coverage and similarity. Cp contigs were assembled based on cp-like reads using SOAPdenovo2 (Luo et al., 2015), then scaffolded by SSPACE (Boetzer et al., 2011). Finally, gaps were filled with clean reads using Gap Filler package (Nadalin et al., 2012). The cp genomes generated from this study are available at GPGD (http://www.gpgenome.com/species/) under species IDs 296, 297, 8797, 16984, 16994, 16995, and 62347 (Liao et al., 2022).

The cp genomes were annotated using CPGAVAS2 (Shi et al., 2019) with default settings, except for taking 2,544 rather than 43 plastomes as the reference dataset. Predicted protein-coding genes were extracted and blasted against Swiss-Prot database and then manually corrected in Apollo software (Lewis et al., 2002), from which the latest GFF3 file was used to update the original CPGAVAS2’s prediction. Additionally, tRNA genes were identified by tRNA scan-SE (Lowe and Chan, 2016). The cp genome structure was visualized using Chloroplot software (Zheng et al., 2020). The GC content of the cp genome was calculated by custom R scripts with functions in seqinr (Charif et al., 2005). The distribution of codon usage was investigated using the software CodonW (Sharp and Li, 1986) with RSCU ratios. SSRs (mono-, di-, tri-, tetra-, penta-, and hexanu-cleotide repeats) were detected by MISA (Beier et al., 2017) by setting mono-, di-, tri-, tetra-, penta-, and hexane-nucleotide SSRs to 10, 5, 4, 3, 3, and 3 repeat units, respectively. The long dispersed repeats: including forward (F), palindromic (P), reverse (R), and complement (C) repeats were identified using the online tool REPuter (Kurtz et al., 2001) with default settings. In addition, the tandem repeats (>10 bp in length) were identified using Tandem Repeats Finder program (Benson, 1999), in which the alignment parameters referred to Fan’s (Fan et al., 2018).

The complete cp genomes of Datureae were aligned using MAFFT (Katoh et al., 2019) and compared at cp genome-level by mVISTA (Frazer et al., 2004). The nucleotide diversity (Pi) was calculated by DnaSP with sliding window analysis by setting step size to 200 bp and window length to 800 bp (Rozas et al., 2017). In addition, IRscope was used to evaluate the IR expansion and contraction with the GenBank files (Amiryousefi et al., 2018).

### 2.3 Selective pressure analysis and phylogenetic analyses

We extracted shared non-redundant gene CDS and among 22 cp genomes from Datureae species, and extracted each gene’s CDS pair of one-by-one species combination and aligned them with MAFFT (Katoh et al., 2019). The rate of non-synonymous (Ka) and synonymous (Ks) substitutions and Ka/Ks were then calculated by ParaAT2.0 (Zhang et al., 2012), which is called KaKs_Calculator2.0 (Zhang et al., 2006) with “MA” model. The command that we applied is as follows: “ParaAT.pl -c 11 -h homologs.txt -n CDS -a PEP -p proc -o OUT -k -f axt -m mafft -v”. In addition to 13 cp genomes of Datureae sequenced in this study (**Supplementary Table S1**), nine published cp genomes from NCBI were retrieved for further phylogeny analysis. In details, *Nicandra physalodes* and *Atropa belladonna* were used as an outgroup to construct the phylogenetic tree (**Supplementary Table S4**), among which four more cp genomes (*D. stramonium* var. *tatula*-2, *D. stramonium* var. *tatula*-3, *B. arborea*-2, and *B. aurea*-3) sequenced in this study were added to obtain more reliable phylogenetic relationships. Maximum likelihood (ML) method in RAxML-ng (Kozlov et al., 2019) was used by providing multiple sequence alignment file generated from whole cp genomes with MAFFT (Katoh et al., 2019). For RAxML-ng, the model parameter was set to “GTR+I+G4,” which was chosen by model test planted in RAxML-ng; bs-metric was set to “fbp, tbe;” 100 starting trees (50 random and 50 parsimony-based) were used to pick the best-scoring topology; and bootstrap replicates were set to 1,000. In addition, MrBayes (Huelsenbeck and Ronquist, 2001) was used for Bayesian inference of phylogeny by setting MCMC simulations to 10,000,000 generations, sampling frequency to one out of every 1,000 generations, and heating coefficient to 0.07. The first 25% of the trees were regarded as burn-ins. Two-runs-two-independent analyses starting from different random trees was used to calculate convergence diagnostics on the fly.

### 2.4 Divergence time estimation

The divergence times between lineages were estimated by clocks module in MEGA11 (Tamura et al., 2021), where we applied the RelTime method (Tamura et al., 2012). Theoretical foundation of the RelTime method for estimating divergence times from variable evolutionary rates to the user-supplied phylogenetic tree using the ML method generated by RAxML-ng and general time reversible substitution model with four categories was chosen. Three calibration constraints queried from TimeTree (Kumar et al., 2022) with normal distribution were set, with node Bar_1-Bau_1 (mean=10.1, sd=0.2), Dino-Dme_2 (mean=9.5, sd=0.2), and Dine-Dst_1 (mean=1.71, sd=0.1), respectively; in addition, Abe-Nph node was set as outgroup.

### 2.5 Molecular markers mining for species identification

First, five universal plant DNA barcodes (ITS, ITS2, *mat*K, *rbc*L, and *psb*A*-trn*H) were used for the testing of the identification for Datureae species. Second, specific primer was designed based on highly variable regions of *D. stramonium* and *D. stramonium* var. *inermis* and used for further validation. The PCR reaction system was performed in a total volume of 25 μl that contained 2× Taq PCR Mix of 12.5 µL, forward primer (10.0 µM) of 1.0 µL, reverse primer (10.0 µM) of 1.0 µL, genomic DNA of 2.0 µL (30–100 ng), and added up to 25 µL with ddH_2_O. The primers and conditions for PCR are listed in **Supplementary Table S3**. All the PCR products were sent to Sangon Biotech Guangzhou branch office for sequencing. The bi-directionally sequenced peaks of DNA markers were assembled using the CondonCode Aligner v8.0.1 software (https://www.codoncode.com/aligner). Neighbor-joining (NJ) trees were constructed with 1,000 bootstrap replicates for each marker in MEGA (Tamura et al., 2021) to evaluate its discrimination power, using 50% as a cutoff value for the condensed tree.

## 3 Results

### 3.1 Cp genome features and organizations

**Figure 2:**
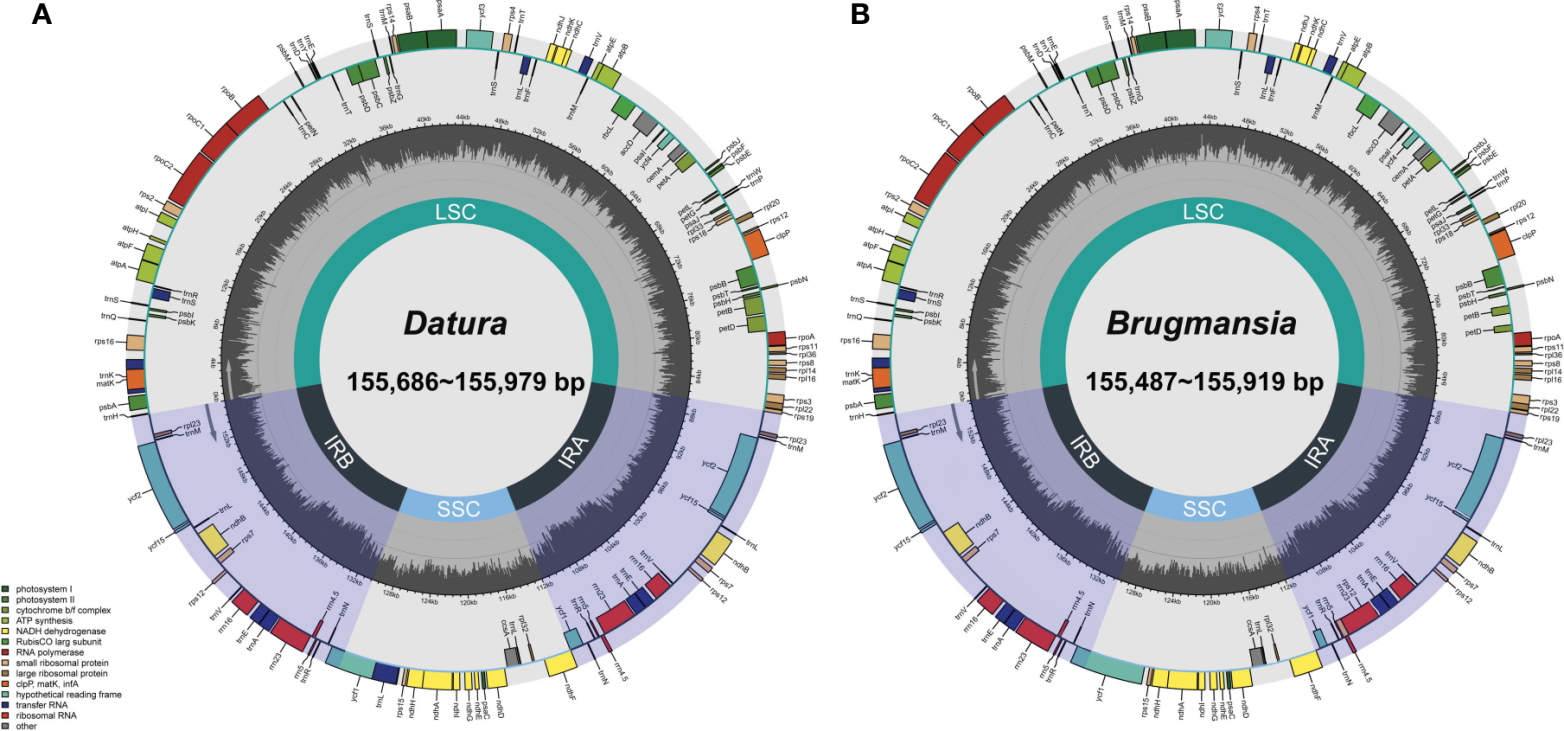
*Datura* **(A)** and *Brugmansia* **(B)** cp genome maps. Genes drawn within the circle are transcribed clockwise; genes drawn outside are transcribed counterclockwise. Genes in different functional groups are shown in different colors. Dark bold lines indicate the extent of IR regions that separate the genomes into SSC and LSC regions.

Fresh leaves of seven Datureae taxa were extracted with total DNA and subjected to NGS with Illumina NovaSeq paired-end sequencing. Cp-like sequences were extracted from clean Illumina reads by BLAST searches against an in-house constructed chloroplast database. As a result, the cp genomes showed typical quadripartite structures, which were divided into LSC region and SSC region by IRa/IRb regions with genome size ranging from 154 bp, 686 bp to 155 bp, 979 bp (**Figure 2**). The overall GC content for these seven taxa was nearly identical (~37.8%) but was unevenly distributed in the cp genomes (**Table 1**). In details, the GC content was the highest in IR regions (approximately 43.0%) while the lowest in SSC regions (approximately 32.0%), and 35.80%–36.02% for LSC regions in both genera. After annotation, 128–132 genes including 83–87 protein-coding genes, 8 rRNA, and 37 tRNA genes were predicted in Datureae species (**Supplememtary Table S5**). Among these genes, 18 duplicated genes were found in the IR region, including eight protein-coding, six tRNA, and four rRNA genes (**Table S5**). We found that *D. metel* and *D. stramonium* var. *tatula* have one more copy of *rps*19 gene than other species, while there is a lack of a copy of *ycf*1 gene in *D. metel*-1. Furthermore, *pet*B and *pet*D genes in *D. stramonium*, *D. stramonium* var. *inmeris*, and two samples of *D. metel* contained one intron for each. Moreover, *pet*B in *D. inoxia* also contained one intron. However, there is no intron in *pet*B or *pet*D genes of *Brugmansia* species (**Supplementary Table S5**).

**Table 1:** Chloroplast genome features of seven Datureae taxa.

Species	Length (bp)				No. of genes				GC content (%)				
	Total	LSC	IR	SSC	Total	Protein-coding genes	rRNA genes	tRNA genes	Total	LSC	Ira	IRb	SSC
*Datura stramonium*	155,896	86,324	18,366	51,206	131	86	8	37	37.87	35.95	43.1	43.1	32.32
*Datura stramonium* var. *inermis*	155,894	86,322	18,366	51,206	131	86	8	37	37.87	35.95	43.1	43.1	32.32
*Datura stramonium* var. *tatula-*1	155,791	85,854	18,495	51,442	132	87	8	37	37.81	35.88	43.08	43.08	32.11
*Datura stramonium* var. *tatula*-2	156,124	86,658	50,997	18,374	130	85	8	37	37.79	35.84	43.15	43.15	32.17
*Datura stramonium* var. *tatula*-3	155,653	88,094	51,035	18,221	128	83	8	37	37.80	35.95	43.12	43.12	32.09
*Datura inoxia*	155,979	86,602	18,353	51,024	131	86	8	37	37.83	35.91	43.1	43.1	32.25
*Datura metel-*1	155,913	86,348	23,101	46,464	131	86	8	37	37.80	35.86	43.42	43.42	33.73
*Datura metel-*2	154,686	85,482	23,218	45,986	132	87	8	37	37.79	35.8	43.48	43.48	33.76
*Brugmansia arborea-*1	155,919	86,277	23,024	46,618	131	86	8	37	37.84	35.91	43.44	43.44	32.7
*Brugmansia arborea*-2	155,964	88,094	49,649	18,221	129	84	8	37	37.84	36.02	43.29	43.29	32.10
*Brugmansia aurea-*1	155,497	86,258	19,129	50,110	131	86	8	37	37.81	35.88	43.31	43.31	32.18
*Brugmansia aurea-*2	155,639	86,089	18,234	51,316	131	86	8	37	37.82	35.89	43.11	43.11	32.05
*Brugmansia aurea*-3	155,630	86,131	49,492	20,007	131	86	8	37	37.82	35.88	43.49	43.49	32.23

### 3.2 Codon usage and repeat analysis

Codon usage patterns and nucleotide composition help to lay a theoretical foundation for genetic modifications of the cp genome (Romero et al., 2000; Angellotti et al., 2007). Amino acids frequency and codon usage were determined in this study, a range of 26, 207–26, and 358 codons were included in the protein-coding genes wherein leucine was the most abundant (10.6%) while cysteine was the least (1.2%) (**Supplementary Figure S1**).

Simple sequence repeats (SSRs) are tandem repeats of DNA sequences with 1–6 bp in length that have been widely applied as molecular markers in species authentication (Powell et al., 1995; Song et al., 2014). In total, 53–59 SSRs were detected among nine cp genomes, among which, mono-nucleotide repeats were the most frequent, followed by di-, tetra-, and tri-repeats. Noteworthy, penta-repeats were only found in *B. arborea* and *B. aurea*, while hexa-repeats were only found in *D. stramonium* var. *tatula*, *D. inoxia*, and *D. metel* with the least numbers (**Supplementary Figure S2A**). Among these SSRs, A/T repeats were the most frequent, followed by AT/AT repeats (**Supplementary Figure S2B**), which were consistent with the majority reported angiosperms (Gao et al., 2019; Li et al., 2019). Except for SSRs, a total of 98–99 long dispersed repeats were detected in nine cp genomes by REPuter (Kurtz et al., 2001), among which the forward and palindrome repeats were found to be the most abundant repeats while complement repeats were the least (**Supplementary Figure S3A**) and repeats with 11–20 bp were most frequent and 41–51 bp were the least among nine cp genomes (**Supplementary Figure S3B**). Additionally, 22–30 tandem repeats were identified in nine cp genomes using Tandem Repeats Finder program (Benson, 1999), in which most of the repeat units were 11–30 bp in length (**Supplementary Figure S3C**).

### 3.3 Boundary regions and comparative analysis

**Figure 3:**
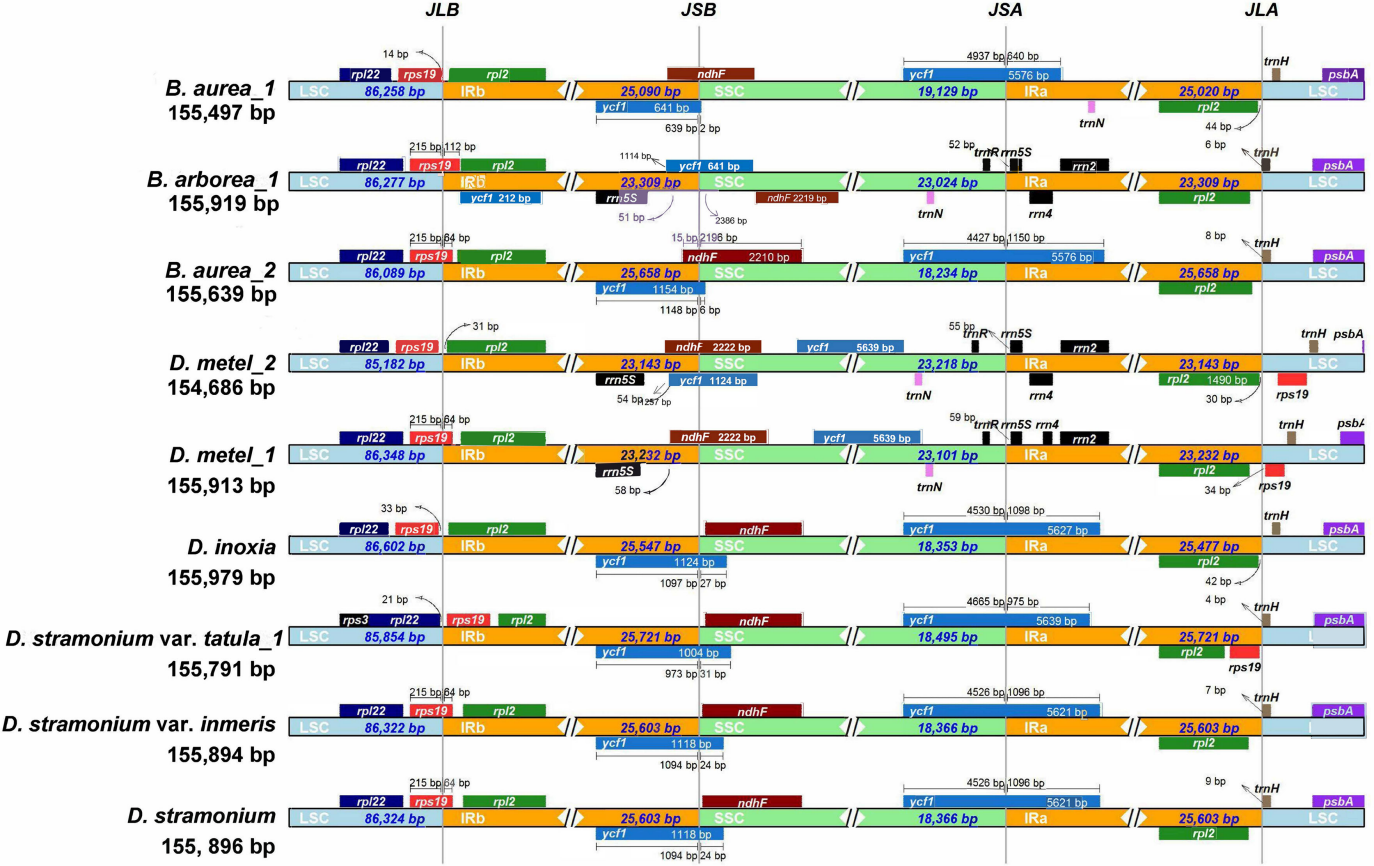
Contraction and expansion of IR borders of Datureae cp genomes.

The expansion and contraction of IR regions are common phenomena during the evolution and the main reason for variations in cp genome length. Therefore, the IRs and SC borders were detected among nine cp genomes. The border of LSC/IRb junction (JLB) located in *rps*19 gene in *D. metel*_1, *D. stramonium*, *D. stramonium* var. *inermis*, *B. arborea*_1, and *B. aurea*_2, but varied 64–112 bp in length overlapping with IRb. In *B. aurea*_1, *D. metel*_2, and *D. inoxia*, *rps*19 gene located in LSC region with 14–332 bp from LSC/IRb border, while in *D. stramonium* var. *tatula*, *rps*19 gene completely contracted into IRb region. To the border of SSC/IRa junction (JSA), except for *B. arborea*_1, *D. metel*_1, and *D. metel*_2, the IRa of other species expanded into *ycf*1 gene. The border of SSC/IRb junction (JSB) was located between *ycf*1 and *ndh*F genes, and JSB of *B. aurea*_2 was located in the overlap of these two genes, while in *B. arborea*_1, two *ycf*1 genes were located in IRb and SSC, respectively. Gene *trn*H located on the border of LSC/IRa was 4–157 bp away from IRa region (**Figure 3**).

**Figure 4:**
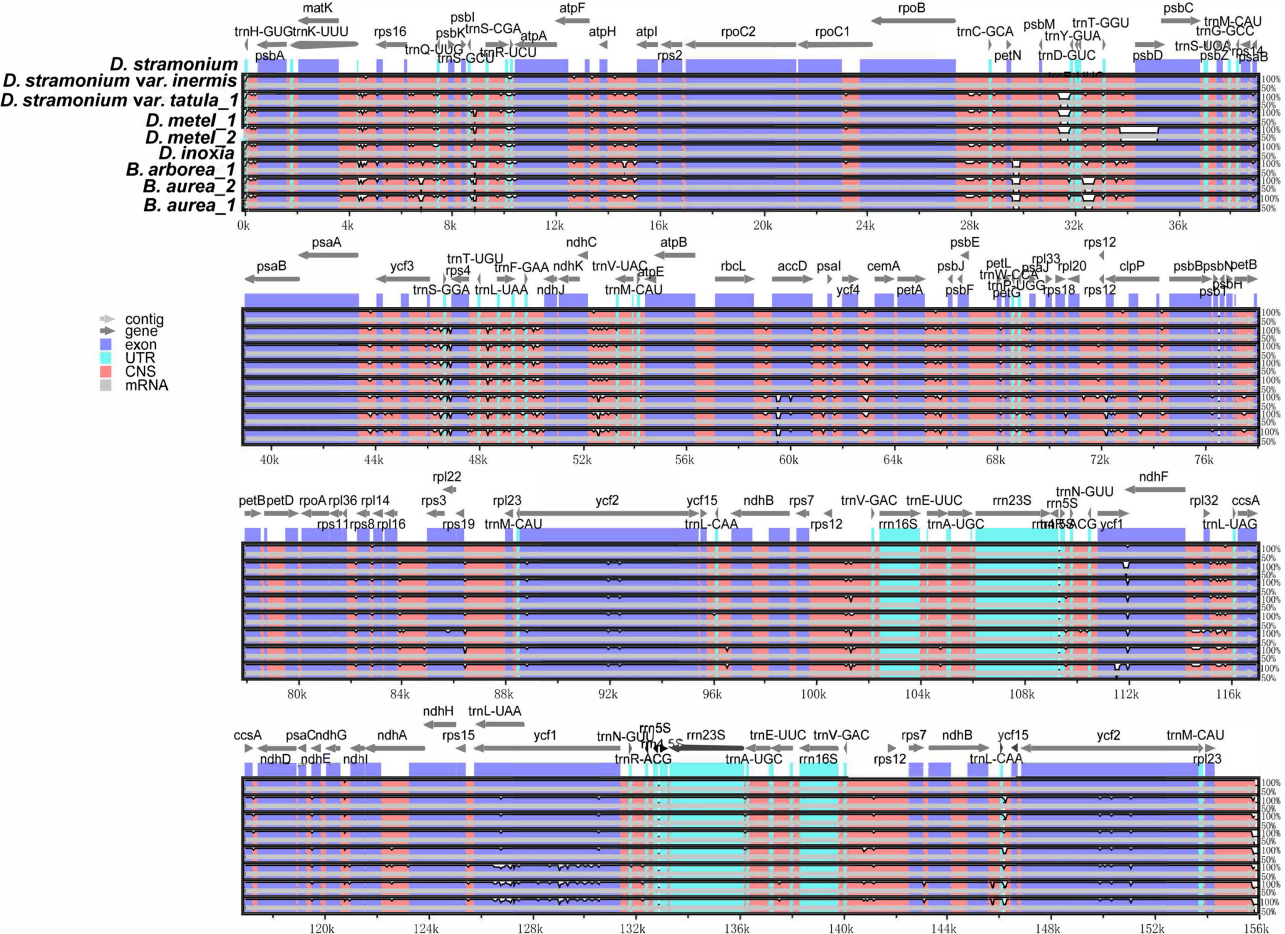
Sequence alignments of Datureae cp genomes using mVISTA with *D. stramonium* as a reference. Gray arrows and thick black lines above the alignment indicate genes with their orientation and the position of the inverted repeats (IRs), respectively. A cutoff of 70% identity was used for the plots, and the Y-scale represents the percent identity ranging from 50% to 100%.

Multiple alignments of cp genomes were conducted taking *D. stramonium* as a reference. The results indicated that gene or sequence order and organization were conserved among these cp genomes. Sequences in non-coding regions were more divergent than coding regions (**Figure 4**). In non-coding regions, the most divergent regions were mainly located in the intergenic spacers. Furthermore, the nucleotide diversity (Pi) values of cp genomes were calculated using DnaSP. Based on DNA polymorphisms, five highly diverged regions were identified, including *ycf*1*_trn*N*-*GUU, *ccs*A*-ndh*D, *rps*16*-psb*K, *atp*H*-atp*I, and *ycf*1, and the Pi value of these regions ranged from 0.1673 (*atp*H*-atp*I) to 0.02932 (*ycf*1*_trn*N*-*GUU), which might be potential candidates for Datureae identification (**Supplementary Figure S4**).

### 3.4 Phylogenetic analysis and selective pressure analyses

**Figure 5:**
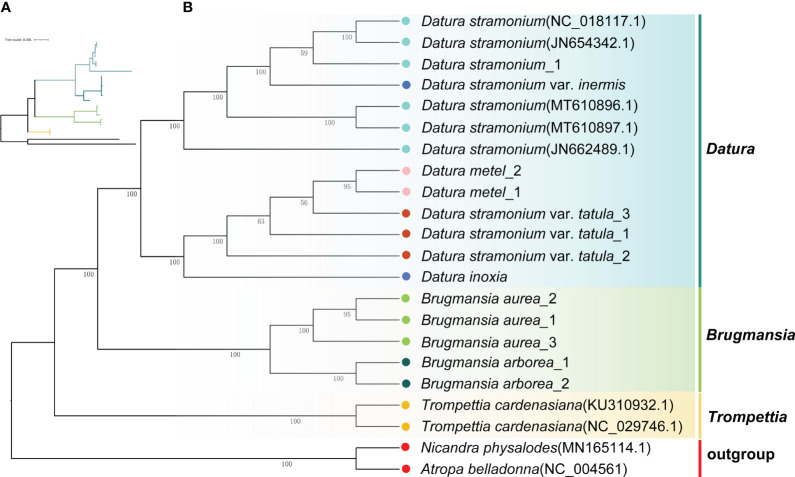
Phylogenetic tree inferred from ML analysis. **(A)** The tree topology of Datureae species. **(B)** Complete ML tree using the cp genomes of 20 Datureae species. The number above the lines indicates the ML bootstrap values.

To unravel the phylogenetic relationships among Datureae species, the phylogenetic tree was re-constructed based on complete cp genomes and super gene of protein coding genes from the 22 Solanaceae species using ML and Bayesian inference (BI) methods, respectively (**Supplementary Tables S1**, **S4**). The topologies of these trees were nearly consistent (**Figure 5**; **Supplementary Figure S5**). The phylogenetic tree of available Datureae species presented two main clades. One clade comprised *T. cardenasiana*, while the other clade was further divided into two subclades, in which one subclade contained species from *Brugmansia* and another subclade contained species from *Datura*, which revealed the monophyly of these three genera. In *Brugmansia* subclade, *B. aurea* and *B. suaveolens* clustered to two branches separately with strong support (bootstrap value=100%). The *Datura* subclade was separated into two branches, one contained *D. stramonium* and *D. stramonium* var. *inermis* and the other contained *D. inoxia*, *D. metel*, and *D. stramonium* var. *tatula.* In detail, different accessories of one species were always clustered together except for those of *D. stramonium* and *D. stramonium* var. *inermis*, indicating close relationships between these two taxa. Molecular divergence dates of the eight Datureae taxa were computed based on the shared unique cp protein-coding gene sequences (**Supplementary Figure S6**). The diversification of Datureae members could be dated back to approximately 27.08 mya, which is in the late Oligocene periods; the divergence between *Datura* and *Brugmansia* occurred at 24.9 mya, which is in the early Neogene periods, suggesting a late Oligocen origin for these two genera within the tribe.

**Figure 6:**
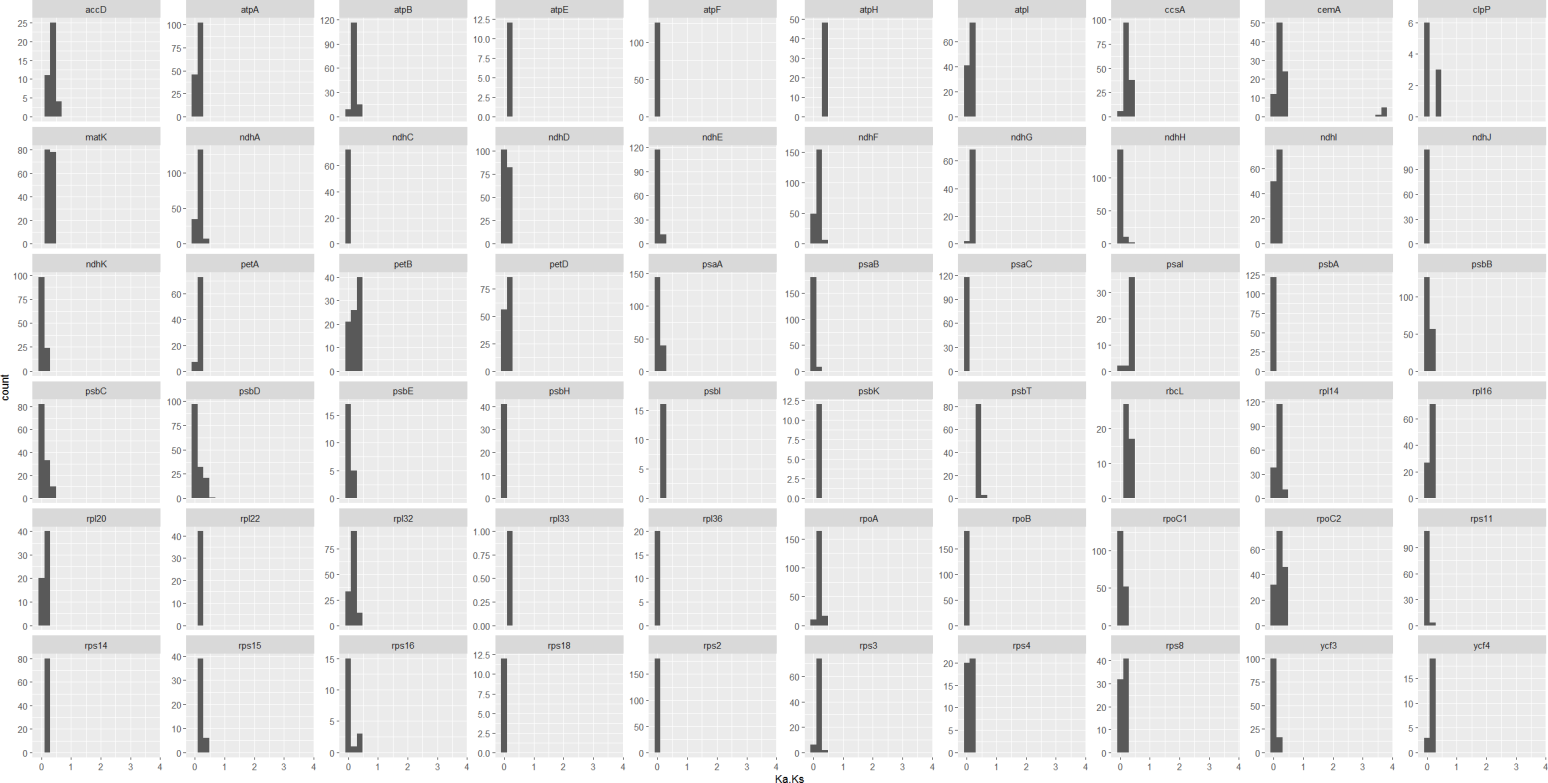
Pairwise Ka/Ks for shared non-redundance from Datureae and genes with >1 substitutions and significant different Ka and Ks values, examined by Fisher’s exact test by KaKs_calculator, were plotted. Genes like *rps*16 only have approximately 20 Ka/Ks values indicating that most of them among species are too conserved to calculate out the Ka/Ks values.

In addition, we calculated the non-synonymous (Ka) and synonymous (Ks) substitution ratios (Ka/Ks) for all the shared unique 69 protein coding genes of cp genomes from 20 Datureae and 2 outgroup species from Solanaceae family, respectively, with KaKs_calculator by “MA” model and statistically tested by Fisher’s exact test. As a result, 31 genes were identified to be positively selected genes along the lineage; in detail, three genes included in ATP subunits (*atp*B, *atp*E, and *atp*H), nine genes in photosystem subunit (*psa*A, *psa*I, *psa*J, *psb*B, *psb*C, *psb*F, *psb*J, *psb*K, and *psb*T), four genes in ribosome large subunit (*rpl*14, *rpl*16, *rpl*32, and *rpl*33), six genes in ribosome small subunit (*rps*3, *rps*4, *rps*11, *rps*15, *rps*16, and *rps*18), three genes in NAD(P)H dehydrogenase complex (*ndh*E, *ndh*G, and *ndh*I), and *clp*P, *pet*B, *rbc*L, *rpo*C1, *ycf*4, and *cem*A genes. However, only one substitution for those genes were detected in the multiple sequence alignment (MSA) files except for *cem*A (chloroplast envelope membrane protein), which owned >20 substitutions in the MSA file. It is noteworthy to mention that there are only ~9.4% of gene pairs, and ~10.0% (after excluding genes with one substitution) are with Ka/Ks >1. Considering that the essence that cp genes are functionally conserved genes and non-synonymous variation is not preferable, we just visualized Ka/Ks for genes with >1 substitutions (**Figure 6**). Overall, Ka/Ks values were <0.5 for the majority genes, suggesting that cp genes of the Datureae species are conserved and mainly under a purifying selection during the evolution process, which is reasonable for necessary functions played by the chloroplast genes and is in accordance with previous studies (Liu et al., 2020).

### 3.5 Mining of molecular marker for species identification based on hotspot region of cp genomes

**Figure 7:**
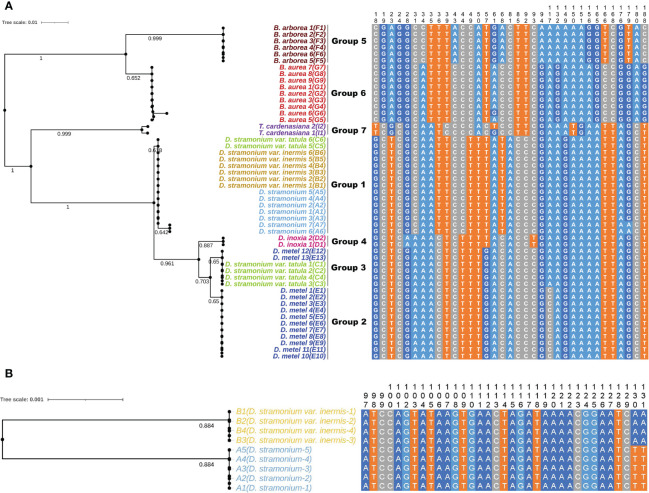
The NJ trees and sequence alignment based on ITS2 **(A)** and the mined marker **(B)** for Datureae species.

Five universal DNA barcodes (ITS, ITS2, *psb*A-*trn*H, *mat*K, and *rbc*L) were amplified and sequenced to assess the identification ability and reveal the phylogenetic relationships among 44 samples of Datureae species (**Figure 7A**; only **Supplementary Figure S7**). The result suggested that universal barcodes were limited in variation detection, and closely related species such as *D. stramonium* and *D. stramonium* var. *inermis* cannot be distinguished from each other. Based on the phylogeny relationship among those species, comparative analysis for genomes sequence was conducted between *D. stramonium* and its variant *D. stramonium* var. *inermis* using sliding window method. As a result, a species-specific site with two mutation sites (TT-AA) was screened out, which were located at 79 bp, 749–79 bp, and 750 bp in the multiple alignment sequence (MSA) file; specific primers for the 330 bp of cp genome region spanned by the mutation site had been tested and validated by PCR amplification and Sanger sequencing (**Figure 7B**; **Supplementary Figure S8**).

## 4 Discussion

### 4.1 Variations in chloroplast genome among Datureae species

The cp genomes ranged in size from 154 bp, 686 bp to 155 bp, 979 bp and from 155 bp, 497 bp to 155 bp, 919 bp for species of *Datura* and *Brugmansia*, respectively. It is a common evolutionary phenomenon in cp genome of plants that the expansion and contraction events of four IR boundaries make the whole cp genome size differ in the same plant population or among different plant populations. As a result, we think that the size variation for these cp genomes may be attributed to the expansion and contraction of the border positions between IR and SC regions (Fan et al., 2018). In addition, repeats composition is another reason responsible for variations in genome size (Chumová et al., 2021; Schley et al., 2022). However, the composition varied little among Datureae species, indicating that expansion and contraction of IR boundaries contribute to the most for cp genome size variation. In most flowering plants, the rearrangement and variation in cp genome sequence are closely related to the frequent variation of repeat regions, mainly caused by unconventional recombination and mismatch (Park et al., 2017). This study analyzed four types of repeat sequences in Datureae species. We found that repeats are similar both in amount and category among Datureae where species clustered in the same branch of the phylogenetic tree have the most similar SSR type and length distribution; the phenomenon is consistent with previous studies for flowering plants (Sugita and Sugiura, 1996). Gene loss-and-gain events is present in some species, despite that cp genomes of land plants are considered to be highly conserved (Daniell et al., 2016). In this study, we found that the number of protein coding gene ranges from 83 to 87 and 84 to 86 in *Datura* and *Brugmansia*, respectively, indicating that gene loss-and-gain event might occurred during the evolutionary process. Furthermore, the Datureae cp genomes have quadripartite structures and conserved gene contents and arrangements, while GC content for IRs is higher than that of LSC and SSC regions. GC content varied among regions, which might attribute to the location of four rRNAs on IRs because rRNA is lacking of AT nucleotides from previous reports (Qian et al., 2013).

### 4.2 Adaptive selection

Synonymous and non-synonymous nucleotide substitution patterns are important markers for gene evolution studies. In most genes, synonymous nucleotide substitutions have occurred more frequently than non-synonymous ones (Ogawa et al., 1999). Thus, a ratio of Ka/Ks < 1 indicates purifying selection, Ka/Ks > 1 denotes probable positive selection, and Ka/Ks values close to one indicate neutral evolution. In this study, the majority of the protein-coding genes of Datureae species were found to be under purifying selection by Ka/Ks analysis, which was conservative in plastid genomes of most angiosperms (Gong et al., 2022). However, we found that 31 cp genes might be under positive selection during the evolution process; these genes are connected to proteins from ATP subunit, photosystem subunit, ribosome large subunit, NAD(P)H dehydrogenase complex, and other genes such as *clp*P, *pet*B, *rbc*L, *rpo*C1, *ycf*4, and *cem*A. These positive selected genes might explain the higher fitness to diverse environment situation for Datureae species. For example, *cem*A plays an important role in protein sorting signals, and it is also be found to undergo adaptation evolution in *Anisodus tanguticus* of Solanaceae and *Gossypium* (Zhou et al., 2022). *psb*T encodes a small hydrophobic polypeptide, which is functional essential to optimize the electron acceptor complex of the acceptor side of PS II (Fagerlund et al., 2020).

### 4.3 Phylogenetic analysis for species in Datureae tribe

The phylogenetic trees constructed with cp genomes indicates that all Datureae species converge into a monophyletic branch, which is divided into three branches with high support for *Datura*, *Brugmansia*, and *Trompettia*. *Trompettia* was previously thought as a sister genus of *Brugmansia* (Särkinen et al., 2013), but in recent years, it was regarded as a common sister genus of *Brugmansia* and *Datura* (Ng and Smith, 2016; Dupin and Smith, 2018) The result was convinced by our study by introducing both universal DNA barcodes and cp genomes. In *Datura*, there are two major sections defined: sect. Datura and sect. Dutra (Bye and Sosa, 2013). Two branches of this genus in our phylogenetic trees match well with this definition. The similar topologies obtained based on various analyses, including these obtained in this study, indicate the clear phylogenetic relationships in Datureae. In FRPS and Flora of China, only one genus *Datura* of this tribe was included. There are four *Datura* species listed in FRPS; they are *D. arborea*, *D. innoxia*, *D. metel*, and *D. stramonium*. The monophyly of *Datura* and *Brugmansia* was once disputed based on morphological characters (Persoon, 1805; Bernhardi, 1833; Safford, 1921; Barclay, 1959), and *B. arborea* was listed as *D. arborea* in FRPS. However, multiple phylogenetic studies supported the separating genera (Bye and Sosa, 2013; Särkinen et al., 2013; Ng and Smith, 2016). Thus, in flora of China, *D. arborea* was removed, and only three species were left. We support this revision according to this and previous phylogenetic studies. However, the opinion described in FRPS that the variation in flower color and seed texture among *D. inermis*, *D. tatula*, and *D. stramonium* might attribute to genetically unstable and evolutionarily meaningless dominant and recessive genes was not fully supported in our study. We supported the hypothesis that *D. inermis* might be regarded as *D. stramonium*, but *D. tatula* was different from *D. stramonium*. In addition, we have estimated the divergence times of eight Datureae taxa based on the protein-coding sequences in the complete cp genomes. The origin time of Datureae tribe was estimated approximately 27.08 mya in Oligocene, which is consistent with the time queried from TimeTree database (a median of 28.6 mya). However, the divergence time estimated in in this study differed from that reported by Dupin and Smith (2018) slightly. Future phylogenetic work in Datureae would benefit from more extensive sampling with cp genomes analysis to resolve uncovered regions of the phylogeny and to provide a robust test for the diversification of the genus.

### 4.4 Species identification for Datureae species

The accurate identification of plant species is important not only for taxonomy but also in agriculture and pharmaceuticals. Generally, ITS2 is an efficient DNA barcode for species identification and have been applied to authenticate for herb extensively (Chen et al., 2023); our study for species identification with ITS2 and other four universal DNA barcodes failed to resolve the identification issues for several closely related Datureae species even with our custom database, which contains 1,276 barcodes (Gong et al., 2018).

Using cp genomes as super barcodes, different accessories of one species clustered together with higher supports than that of ITS2, suggesting that cp genome could be a super barcode with potential in the authentication of Datureae species. However, *D. stramonium* and *D. stramonium* var. *inermis* still could not be discriminated between each other, indicating close relationships between these two taxa. Intergenic regions play important roles in gene expression regulation and can accumulate more mutations than protein-coding regions, and therefore, they can be used to develop molecular markers for reconstruction of phylogeny (Parenteau and Abou Elela, 2019). Based on the hotspots of variation between *D. stramonium* and *D. stramonium* var. *inermis*, we have developed a molecular marker and succeeded in distinguishing these closely related species. However, it is worth mentioning that we found that *D. stramonium* var. *tatula*, supposed to be relatives to *D. stramonium*, was closer to *D. metel*, which is inconsistent with a previous report (Wu et al., 2015). Taking the uniparental inheritance characteristics of cp genome and similarity in morphological traits, we hypothesized that *D. stramonium* var. *tatula* might be a hybrid of *D. stramonium* and *D. metel*; however, substantial evidence still needs to be provided.

## 5 Conclusion

In this study, we have sequenced, *de novo* assembled and annotated 13 complete cp genomes of seven taxa from Datureae tribe. Variations in genome size, repeat composition, gene composition, selective pressure, and evolution trajectory among them were studied. A total of 31 genes identified as positive-selected genes during evolution might play important function roles in the adaptive process. In addition, we succeeded in distinguishing *D. stramonium* from its closely related species *D. stramonium* var. *inermis* by screening out molecular markers based on the high variation hotspots that were resulted from comparative analysis at cp genomic level. These cp genomes are useful genetic resources for taxonomy, phylogeny, and evolution for Datureae.

## Data availability statement

The data presented in the study are deposited in the Global Pharmacopoeia Genome Database (http://www.gpgenome.com/species/) under species IDs 296, 297, 8797, 16984, 16994, 16995 and 62347.

## Author contributions

HS: Methodology, Software, Writing – original draft. XD: Writing – original draft, Data curation, Formal Analysis. BL: Data curation, Formal Analysis, Visualization, Writing – original draft. DZ: Visualization, Writing – original draft. JH: Supervision, Writing – original draft. JB: Supervision, Writing – review & editing. SX: Validation, Writing – review & editing. JZ: Project administration, Writing – review & editing. WX: Writing – review & editing, Project administration. XQ: Investigation, Validation, Writing – review & editing. LG: Investigation, Validation, Writing – review & editing. ZH: Project administration, Writing – review & editing, Conceptualization, Funding acquisition, Resources.
