# Supplementary material for: Comparative chloroplast genomes provided insights into the evolution and species identification on the Datureae plants
Source: Front Plant Sci. 2023 Oct 24;14:1270052. doi: 10.3389/fpls.2023.1270052 (PMC10628451; doi:10.3389/fpls.2023.1270052)
Supplement: Supplementary file 1 [file DataSheet_1.docx]

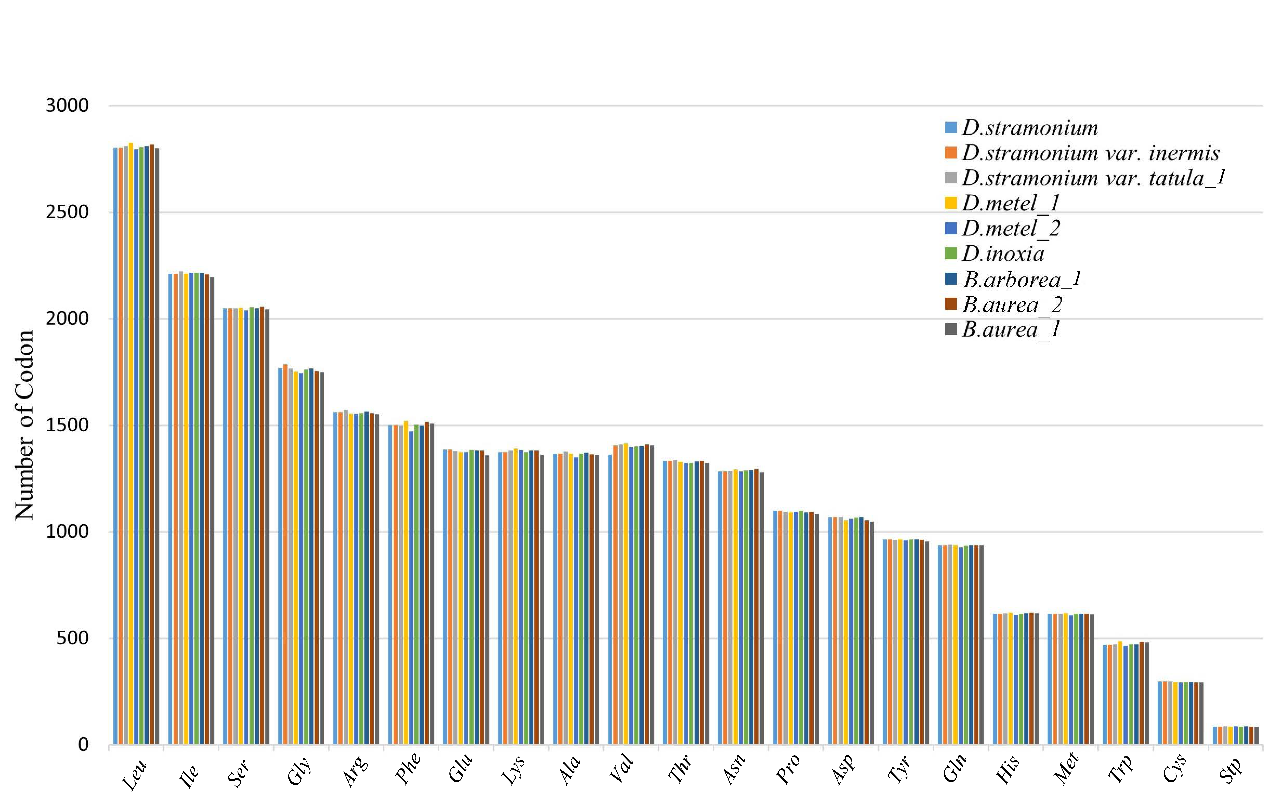
Figure S1 Number of Codon for the 20 amino acids and stop codons of CDS in nine Datureae cp genomes**.**

**
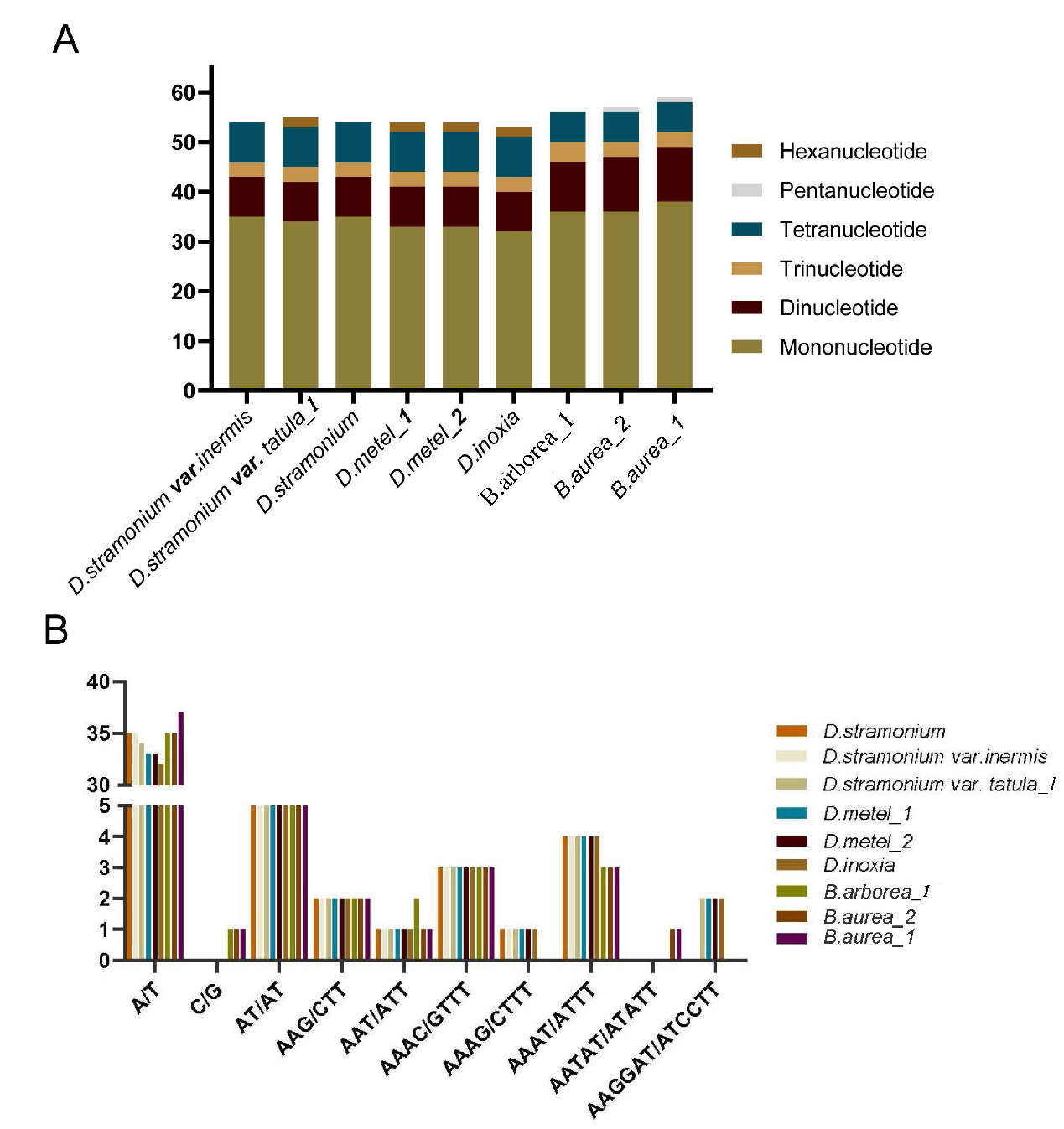
**Figure S2 Analysis of type and motifs of SSRs in nine Datureae cp genomes. A, Analysis of simple sequence repeats (SSRs) in the cp genomes of nine Datureae species. B, Frequency of identified SSR motifs in different repeat class types.

**
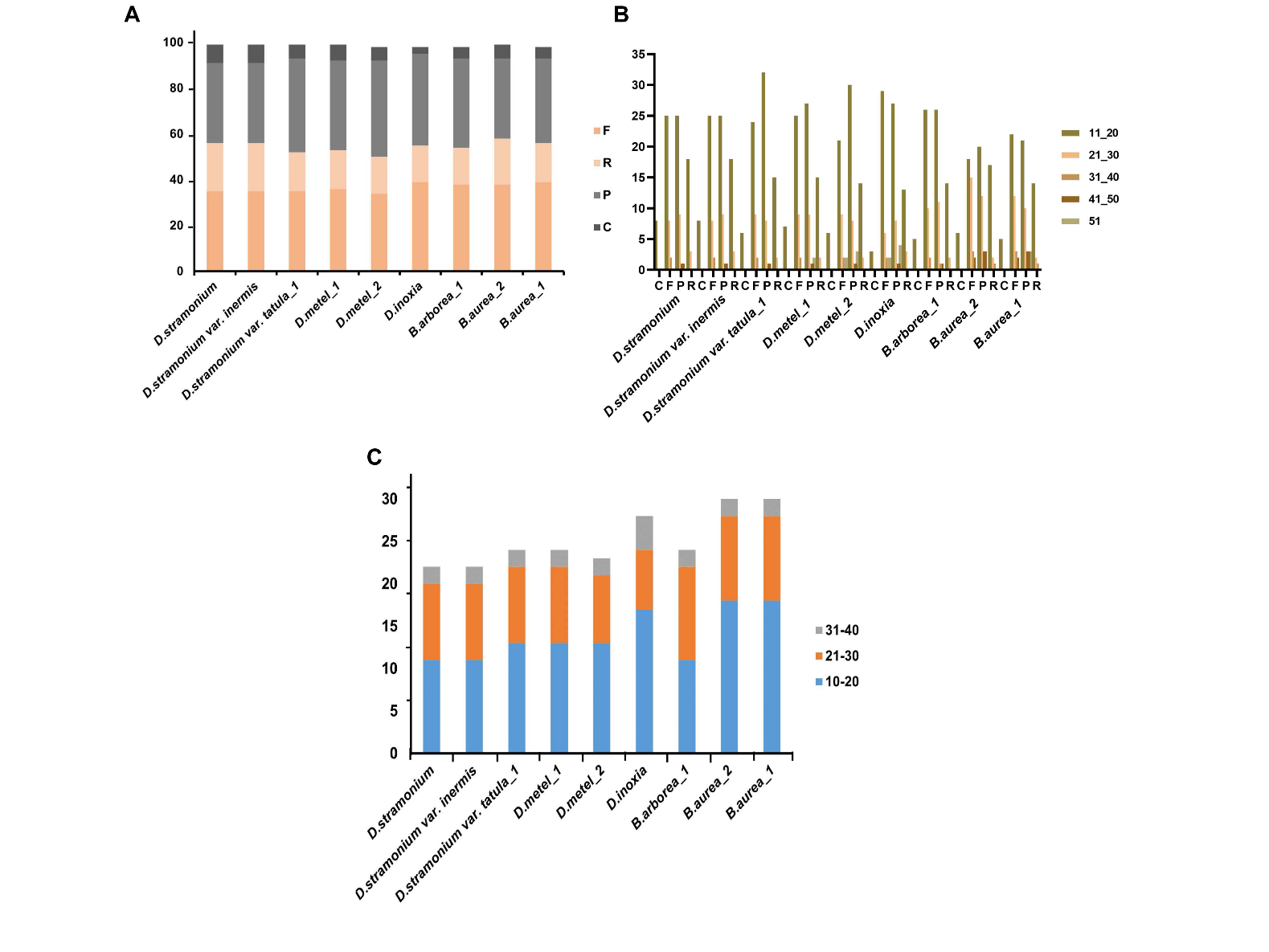
**

Figure S3 Long repeats analysis of nine Datureae cp genomes. A, Number of long dispersed repeats in nine Datureae cp genomes. B, Number of different types of dispersed repeats. F, P, R, and C indicate the repeat types F (forward), P (palindrome), R (reverse), and C (complement), respectively. C, Number of tandem repeats in nine Datureae cp genomes.

**
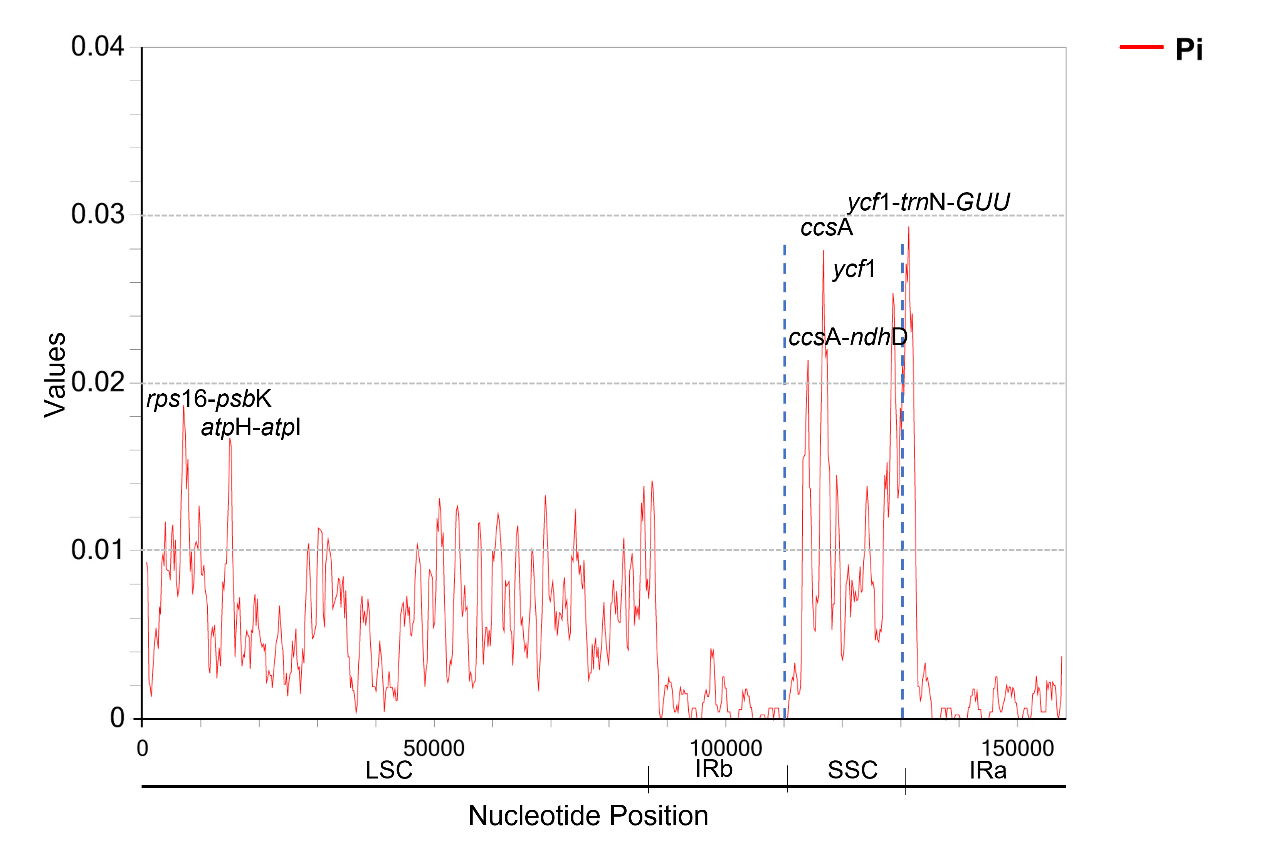
**

Figure S4 Sliding window analysis of nine Datureae cp genomes. Window length: 800 bp; step size: 200 bp. X-axis: position of the midpoint of a window. Y-axis: nucleotide diversity of each window.


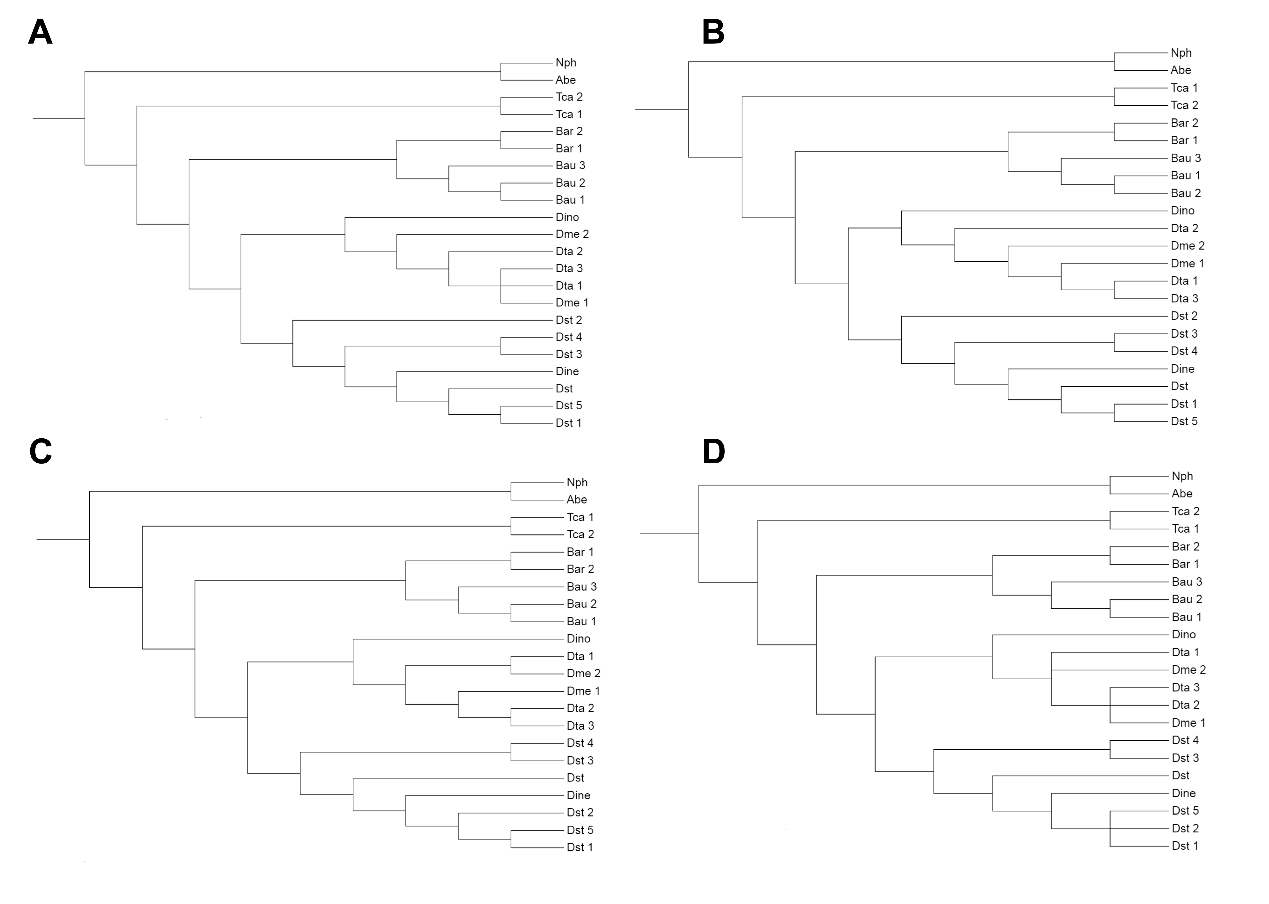


Figure S5 ML trees and BI trees generated by RAxML-ng and MrBayes respectively. (A), Phylogenetic tree constructed with whole cp genome by ML method. (B), Phylogenetic tree constructed with super gene concatenated of shared non-redundant protein coding genes by ML method. (C), Phylogenetic tree constructed with super gene concatenated of shared non-redundant protein coding genes by BI method. (D) Phylogenetic tree constructed with whole cp genome by BI method.


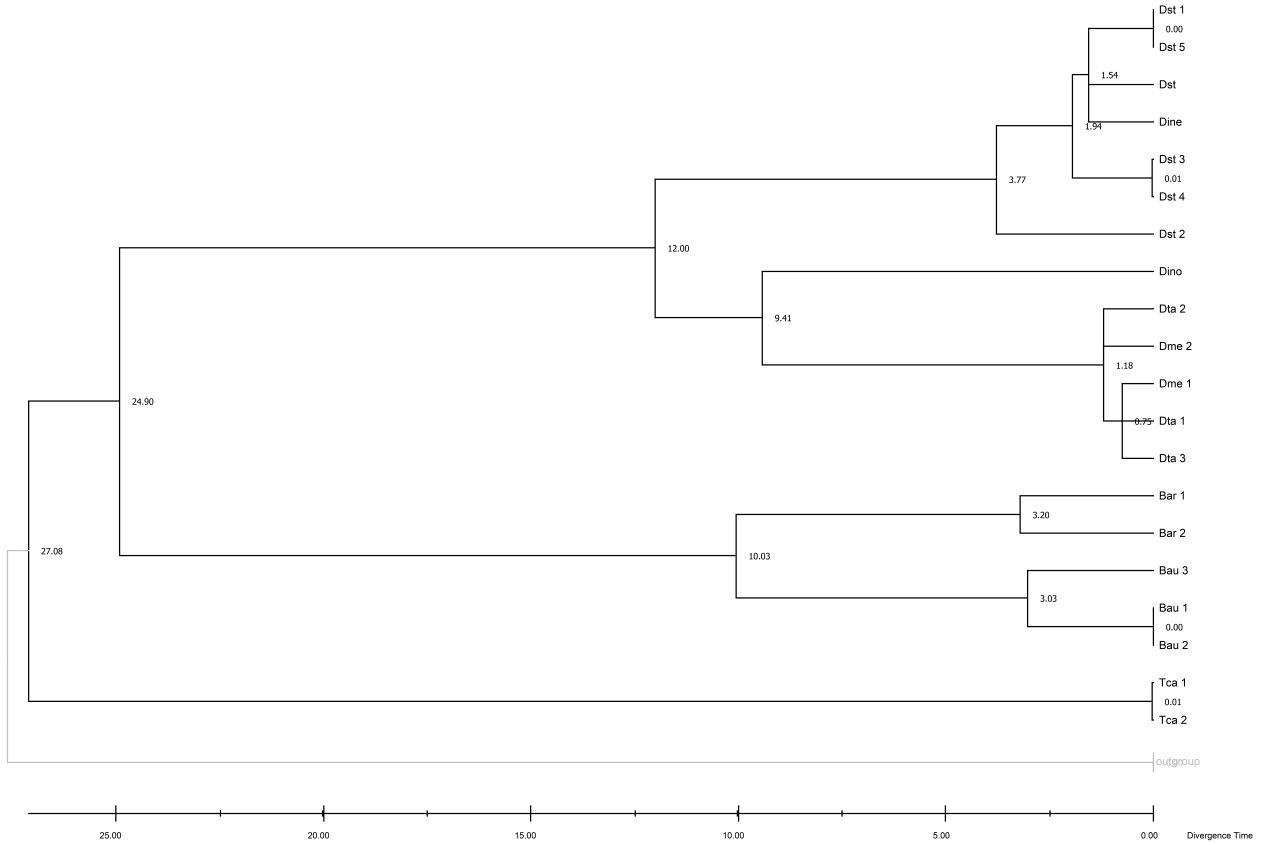


Figure S6 Molecular dating of 22 Datureae species based on the shared non-redundant protein-coding genes in cp genomes.


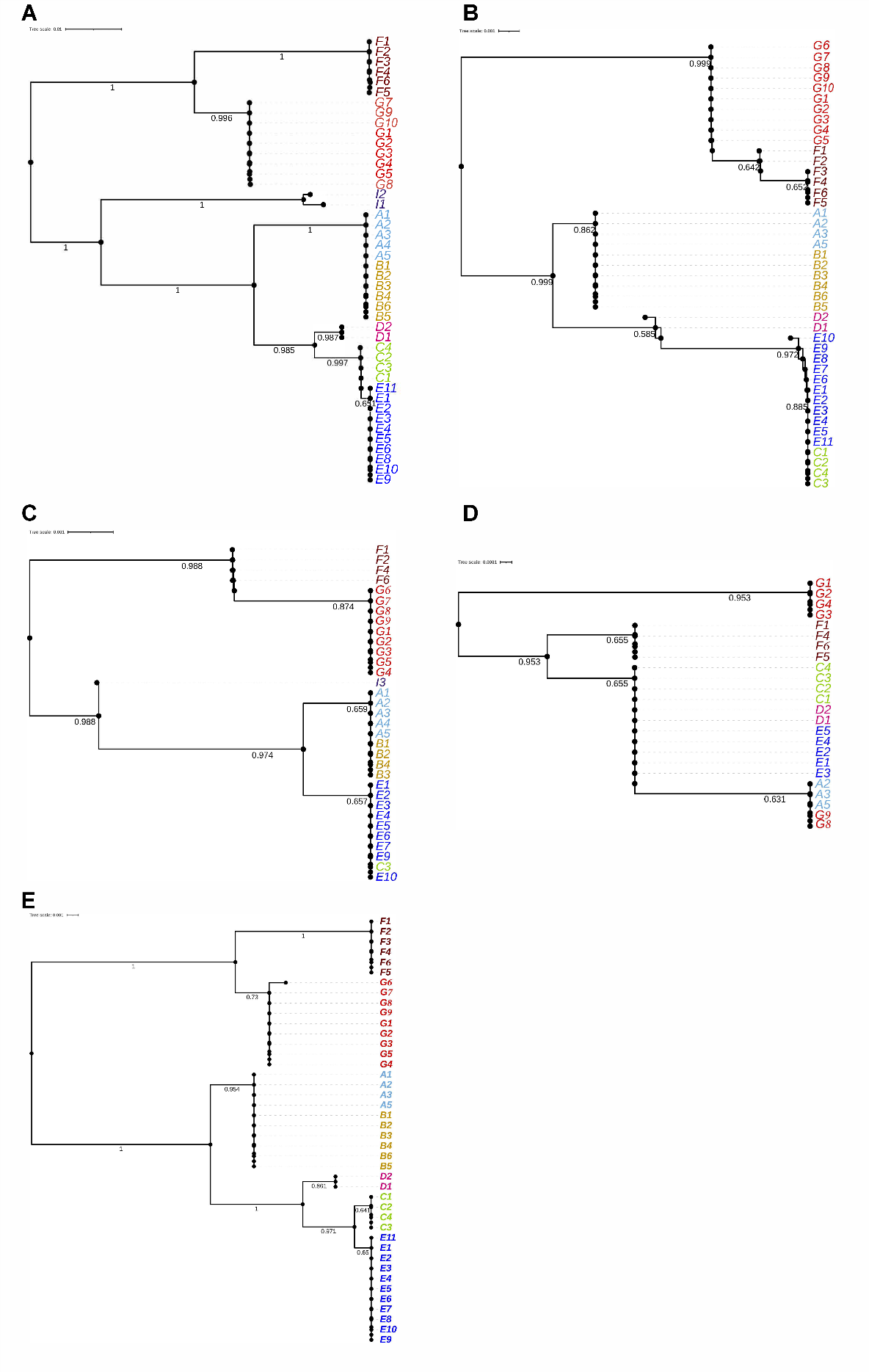


Figure S7 The NJ trees constructed with ITS (A)、*psb*A-*trn*H (B)、*mat*K (C)、*rbc*L (D)and ITS2+*psb*A-*trn*H（E）for Datureae species.

**
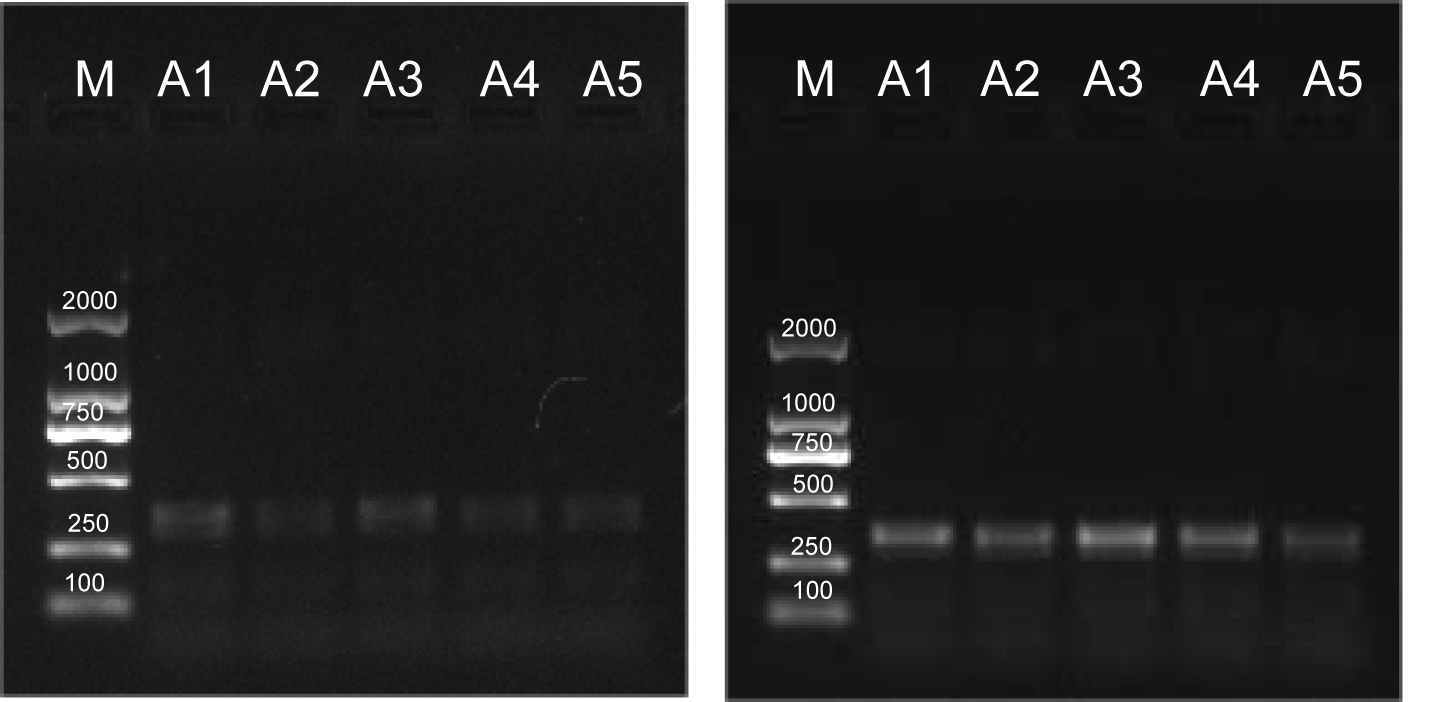
**

Figure S8 Electropherogram of PCR products with specific primers for *D. stramonium* (A1-A5) and *D. stramonium* var *inermis* (B1-B5).
