# Supplementary material for: Comparative chloroplast genomes provided insights into the evolution and species identification on the Datureae plants
Source: Front Plant Sci. 2023 Oct 24;14:1270052. doi: 10.3389/fpls.2023.1270052 (PMC10628451; doi:10.3389/fpls.2023.1270052)
Supplement: Supplementary file 2 [file DataSheet_2.docx]

Table S1 Samples information for high throughput sequencing.

| **Sample ID** | **Sampling Site** | **Identifier** | **Note** | **Voucher ID** |
| --- | --- | --- | --- | --- |
| *Datura stramonium* | Huanggang City, Hubei Province | Chongjian Zhou |  | Dst |
| *Datura stramonium* var. *inermis* | Huanggang City, Hubei Province | Chongjian Zhou |  | Dine |
| *Datura stramonium* var. *tatula*-1 | Huanggang City, Hubei Province | Chongjian Zhou |  | Dta1 |
| *Datura stramonium* var. *tatula*-2 | Wuhan City, Hubei Province | Chongjian Zhou | For validation in phylogenetic analysis | Dta2 |
| *Datura stramonium* var. *tatula*-3 | Wuhan City, Hubei Province | Chongjian Zhou | For validation in phylogenetic analysis | Dta3 |
| *Datura inoxia* | Huanggang City, Hubei Province | Chongjian Zhou |  | Dino |
| *Datura metel*-1 | Guangzhou City, Guangdong Province | Huagu Ye |  | Dme1 |
| *Datura metel*-2 | Huanggang City, Hubei Province | Chongjian Zhou |  | Dme2 |
| *Brugmansia arborea*-1 | Guangzhou City, Guangdong Province | Jizhu Liu |  | Bar1 |
| *Brugmansia arborea*-2 | Huanggang City, Hubei Province | Chongjian Zhou | For validation in phylogenetic analysis | Bar2 |
| *Brugmansia aurea*-1 | Huanggang City, Hubei Province | Chongjian Zhou |  | Bau1 |
| *Brugmansia aurea*-2 | Guangzhou City, Guangdong Province | Huagu Ye |  | Bau2 |
| *Brugmansia aurea*-3 | Huanggang City, Hubei Province | Chongjian Zhou | For validation in phylogenetic analysis | Bau3 |

Table S2 Samples information for DNA barcoding and specific primers ming.

| **Code** | **Species** | **Sampling Site** | **Identifier** |
| --- | --- | --- | --- |
| A1-A5 | *Datura stramonium* | Huanggang City, Hubei Province | Chongjian Zhou |
| B1-B6 | *Datura stramonium* var. *inermis* | Huanggang City, Hubei Province | Chongjian Zhou |
| C1-C4 | *Datura stramonium* var. *tatula* | Huanggang City, Hubei Province | Chongjian Zhou |
| D1-D2 | *Datura inoxia* | Huanggang City, Hubei Province | Chongjian Zhou |
| E1-E5 | *Datura metel* | Huanggang City, Hubei Province | Chongjian Zhou |
| E6-E10 | *Datura metel* | Guangzhou City, Guangdong Province | Jizhu Liu |
| F1-F5 | *Brugmansia arborea* | Huanggang City, Hubei Province | Chongjian Zhou |
| F6 | *Brugmansia arborea* | Guangzhou City, Guangdong Province | Jizhu Liu |
| G1-G5 | *Brugmansia aurea* | Huanggang City, Hubei Province | Chongjian Zhou |
| G6-G7 | *Brugmansia aurea* | Guangzhou City, Guangdong Province | Huagu Ye |
| G8-G10 | *Brugmansia aurea* | Guangzhou City, Guangdong Province | Jizhu Liu |

Table S3 Primers and PCR conditions information for DNA barcoding and specific primers ming.

| **Gene fragment** | **Primer name** | **Primer sequence** | **PCR condition** |
| --- | --- | --- | --- |
| ITS | 5a fwd | CCTTATCATTTAGAGGAAGGAG | 94℃ 5min; 94℃ 1min, 50℃ 1min, 72℃ 1min+3s, 30 cycles; 72℃,7min |
|  | 4 rev | TCCTCCGCTTATTGATATGC |  |
| ITS2 | S2F | ATGCGATACTTGGTGTGAAT | 94℃ 5min; 94℃ 30s, 56℃ 30s, 72℃ 45s, 40 cycles; 72℃,10min |
|  | S3R | GACGCTTCTCCAGACTACAAT |  |
| *psb*A*-trn*H | fwd PA | GTTATGCATGAACGTAATGCTC | 94℃ 5min; 94℃ 1min, 55℃ 1min, 72℃ 1.5min, 30 cycles; 72℃,7min |
|  | rev TH | CGCGCATGGTGGATTCACAATCC |  |
| *mat*K | 3F_KIM | CGTACAGTACTTTTGTGTTTACGAG | 94℃ 1min; 94℃ 30s, 52℃ 20s, 72℃ 50s, 35 cycles; 72℃ 5min |
|  | 1R_KIM | ACCCAGTCCATCTGGAAATCTTGGTTC |  |
| *rbc*L | 1f | ATGTCACCACAAACAGAAAC | 95℃ 2min; 94℃ 1min, 55℃ 30s, 72℃ 1min, 34 cycles; 72℃ 7min |
|  | 724r | TCGCATGTACCTGCAGTAGC |  |
| Specific primers | F | TTTGTTCCAATCGCCGTGTA | 94℃ 5min; 94℃ 30s, 59℃ 30s, 72℃ 45s, 40cycles; 72℃ 10min |
|  | R | TGGGTCGTCTCAGACCTTCT |  |

Table S4 Cp genomes downloaded from Genbank for phylogenetic tree construction.

| **Species** | **Accession number** | **Abbreviation** |
| --- | --- | --- |
| *Datura stramonium* | MT610897.1 | Dst1 |
| *Datura stramonium* | MT610896.1 | Dst2 |
| *Datura stramonium* | JN654342.1 | Dst3 |
| *Datura stramonium* | JN662489.1 | Dst4 |
| *Datura stramonium* | NC_018117.1 | Dst5 |
| *Trompettia cardenasiana* | NC_029746.1 | Tca1 |
| *Trompettia cardenasiana* | KU310932.1 | Tca2 |
| *Nicandra physalodes* | MN165114.1 | Npy |
| *Atropa belladonna* | NC_004561 | Abe |

Table S5 Gene contents in the cp genomes of Datureae species.

| **Category of genes** | **Group of genes** | **NO.** |  |
| --- | --- | --- | --- |
| photosystem I | *psaA, psaB, psaC, psaI, psaJ* | 5 |  |
| photosystem II | *psbA, psbB, psbC, psbD, psbE, psbF, psbH, psbI, psbJ, psbK, psbM, psbN, psbT, psbZ, ycf3^**^* | 15 |  |
|  |  |  |  |
| cytochrome b/f complex | *petA, petB^*^, petD^*^, petG, petL, petN* | 6 |  |
| ATP synthase | *atpA, atpB, atpE, atpF^*^, atpH, atpI* | 6 |  |
| NADH-dehydrogenase | *ndhA^*^, ndhB(×2)^*^, ndhC, ndhD, ndhE, ndhF, ndhG, ndhH, ndhI, ndhJ, ndhK* | 12 |  |
| rubisco | *rbc*L | 1 |  |
| RNA polymerase | *rpoA, rpoB, rpoC1^*^, rpoC2* | 4 |  |
| Ribosomal protein(small subunit) | *rps11, rps12(×2), rps14, rps15, rps16^*^, rps18, rps19a, rps2, rps3, rps4, rps7(×2), rps8* | 14(15) |  |
| Ribosomal proteins(large subunit) | *rpl*2(×2), *rpl*14, *rpl*16, *rpl*20, *rpl*22, *rpl*23(×2), *rpl*32, *rpl*33, *rpl*36 | 11 |  |
| Proteins of unknown function | *ycf*1(×2)^b^, *ycf*15(×2), *ycf*2(×2), *ycf*4 | 7(6) |  |
| Transfer RNAs | *trnH-GUG, trnK-UUU, trnQ-UUG, trnS-GCU, trnS-CGA,  trnR-UCU, trnC-GCA, trnD-GUC, trnY-GUA, trnE-UUC(×3), trnT-GGU, trnS-UGA, trnG-GCC, trnM-CAU(×4), trnS-GGA, trnT-UGU, trnL-UAA, trnF-GAA, trnV-UAC, trnW-CCA, trnP-UGG, trnL-CAA(×2), trnV-GAC(×2), trnA-UGC(×2), trnR-ACG(×2), trnN-GUU, trnL-UAG, trnN-GUU* | 37 |  |
|  |  |  |  |
|  |  |  |  |
|  |  |  |  |
|  |  |  |  |
| Ribosomal RNAs | *rrn16S(×2), rrn23S(×2), rrn5S(×2), rrn4.5S(×2)* | 8 |  |
| Other genes | *accD, ccsA, cemA, clpP^**^, matK* | 5 |  |

(×2) indicates the gene sequence is repeated twice. ^*^ indicates genes containing one intron; while ^**^ indicates gene containing two introns. ^a^ indicates there are two copies of *rps*19 in two samples of *D. metel* and *D. stramonium* var. *tatula*. ^b^ indicates there is only copy of *ycf*1 in *D. metel*-1.

Table S6 Characteristics of DNA barcodes for Datureae species.

| **DNA barcodes** | **NO. of sequences** | **successful sequencing rates** | **sequence length (bp)** | **variable sites (%)** | **Intraspecific variation** | **Interspecific variation** | **GC content (%)** |
| --- | --- | --- | --- | --- | --- | --- | --- |
| ITS2 | 43 | 97.73% | 230～233 | 42（18.26%） | 0～0.025 | 0.001～0.139 | 64.78 |
| *psb*A*-trn*H | 43 | 97.73% | 461～531 | 18（3.70%） | 0～0.002 | 0～0.027 | 27.53 |
| ITS | 42 | 95.45% | 648～736 | 98（15.63%） | 0～0.022 | 0～0.114 | 60.39 |
| *mat*K | 33 | 72.73% | 701～846 | 11（1.62%） | 0～0.002 | 0～0.015 | 33.78 |
| *rbc*L | 24 | 54.55% | 659～717 | 4（0.62%） | 0～0.002 | 0～0.006 | 43.3 |

| Sample ID | Num_seqs | Sum_len(Gb) | Min_len(bp) | Avg_len(bp) | Max_len(bp) |
| --- | --- | --- | --- | --- | --- |
| *Brugmansia aurea-*3 | 57,289,902 | 8.63 | 30 | 150.6 | 151 |
| *Datura inoxia* | 53,382,190 | 8.04 | 30 | 150.6 | 151 |
| *Datura metel-*2 | 112,081,036 | 16.9 | 30 | 150.8 | 151 |
| *Datura stramonium* var. *tatula-*3 | 30,879,398 | 4.63 | 150 | 150 | 150 |
| *Brugmansia aurea-*1 | 27,858,588 | 4.18 | 150 | 150 | 150 |
| *Brugmansia aurea-*2 | 30,829,118 | 4.62 | 150 | 150 | 150 |
| *Datura stramonium* var. *tatula-*2 | 35,115,498 | 5.27 | 150 | 150 | 150 |
| *Datura stramonium* var. *tatula-*1 | 55,241,300 | 8.34 | 151 | 151 | 151 |
| *Datura stramonium* | 121,086,796 | 18.28 | 151 | 151 | 151 |
| *Datura* *stramonium* var. *inermis* | 57,814,098 | 8.73 | 151 | 151 | 151 |
| *Brugmansia arborea-*1 | 43,141,150 | 6.51 | 151 | 151 | 151 |
| *Datura metel-*1 | 43,887,494 | 6.63 | 151 | 151 | 151 |
| *Brugmansia arborea-*2 | 43,868,386 | 6.62 | 151 | 151 | 151 |

Table S7 Sequceing data of Datureae species.
